# Functional trait plasticity diverges between sexes in African cichlids: A contribution toward ecological sexual dimorphism?

**DOI:** 10.1002/ece3.10702

**Published:** 2023-11-28

**Authors:** Kirsty McWhinnie, Deepti Negi, K. Elizabeth Tanner, Kevin J. Parsons

**Affiliations:** ^1^ Institute of Biodiversity, Animal Health, and Comparative Medicine University of Glasgow Glasgow UK; ^2^ School of Engineering and Materials Science Queen Mary University of London London UK

**Keywords:** cichlid, craniofacial variation, functional morphology, maternal care, mouthbrooding

## Abstract

Phenotypic plasticity enables development to produce multiple phenotypes in response to environmental conditions. Plasticity driven variation has been suggested to play a key role in adaptive divergence, and plasticity itself can evolve. However, the interaction of plasticity with the multiple levels involved with adaptive divergence is less understood. For example, sexual dimorphism can contribute adaptive variation through ecological sexual dimorphism (ESD), but the contribution of plasticity to this phenomenon is unknown. Therefore, to determine the potential contribution of plasticity to ESD, we used the adaptive radiation of Malawi cichlids. Two mouthbrooding species (*Labeotropheus fuelleborni* and *Tropheops* “Red Cheek”) with differences in foraging tactics underwent foraging experiments using benthic and limnetic treatments while accounting for sex. Plasticity in craniofacial shape and three functionally important traits were measured. Plasticity was shown, but without any sex‐based differences in shape. However, for mechanical advantage traits of the mandible sex by diet interactions were found. This suggests that ESD, may be influenced by phenotypic plasticity that diverges between sexes. Given the involvement of the mandible in parental care in cichlids this may indicate that sexual divergence in plasticity may trade‐off against maternal care tactics.

## Graphical abstract

Adaptive divergence can occur between species, but also between sexes. We tested whether plasticity, which is thought to contribute to species divergence, would differ between sexes in morphological and functional traits. We found some support that plasticity differed between sexes suggesting that may play a role in sex‐based adaptive divergence.

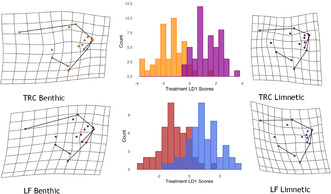

## 1 INTRODUCTION

A fundamental goal of evolutionary biology is to understand how adaptive phenotypic variation arises. Phenotypic plasticity whereby multiple phenotypes can arise in response to environmental conditions has become increasingly viewed as a provider of variation for adaptive divergence (Laland et al., 2015; Parsons et al., 2016; Pfennig et al., 2010; West‐Eberhard, 2003; Wund et al., 2008). While evidence suggests plasticity contributes toward adaptive processes, its influence on different levels of divergence (e.g. species, populations, sexes) is less appreciated. For example, sexual dimorphism is common within vertebrates but contributions toward such dimorphisms from plasticity are not well understood. Related to this is ecological sexual dimorphism (ESD), whereby adaptive divergence evolves between sexes (Shine, 1989), and can be nested within broader patterns of ecological divergence (Foster et al., 1998; Parsons et al., 2015; Riopel et al., 2008). However, an ecological cause for sexual dimorphism is challenging to demonstrate because other causes of sexual dimorphism confound the ecological causes of divergence between sexes in traits, such as size, nutritional requirements, sexual selection and reproductive output (Bolnick & Doebeli, 2003; Shine, 1989; Slatkin, 1984). Nonetheless, there are clear examples of ESD recorded in snakes (Camilleri & Shine, 1990; Houston & Shine, 1993; Vincent et al., 2004), hummingbirds (Temeles et al., 2000, 2010), and Caribbean *Anolis* lizards (Butler et al., 2007; Butler & Losos, 2002). Indeed, in the case of three‐spine sticklebacks (*Gasterosteus aculeatus*), head shape can have minimal overlap between sexes, with adaptive variation associated with sexual dimorphism exceeding the differences between ecological species in some populations (Aguirre & Akinpelu, 2010; Aguirre et al., 2008; Cooper et al., 2011). From these examples, it can be suggested that sexual dimorphism impacts on the interaction of organisms with their environment. While this seems to alter the selection faced by each sex, it should also provide different environmental cues between sexes that alter phenotypic development. Indeed, phenotypic plasticity is widely viewed as a contributor to adaptive divergence, even seen by some to be a necessary source of variation for the initiation of evolutionary change (Levis & Pfennig, 2016). Given that ESD is a form of adaptive divergence, it can be postulated that plasticity contributes to its evolution. Thus, plasticity may evolve sexual dimorphism in systems showing evidence of ESD.

Changes in trophic morphology are key to many cases of ecological adaptation. While ecology can often be inferred from craniofacial morphology, analysis of functionally relevant traits can more precisely assess adaptive responses (including plasticity and ESD) (Aguirre & Akinpelu, 2010; Camilleri & Shine, 1990; Vincent et al., 2004). For example, ecological adaptation along a benthic/limnetic habitat axis is characteristic of many different fishes (Adams & Huntingford, 2002; Cooper et al., 2010; Rundle, 2002; Wainwright, 1996). A steep craniofacial profile with short jaws provides a more benthic phenotype as it confers an advantage for powerful bites, whereas a gradually sloping profile with long jaws facilitates the fast movements required for suction feeding in a pelagic habitat (Cooper et al., 2010). Adaptation to these habitats is often assessed through direct functional assessments of feeding performance that are correlated with diet and prey use (Wainwright, 1996). Alternatively, measurements from relevant anatomical traits can be used indirectly to infer functional performance based on biomechanical principals (Wainwright & Richard, 1995).

In fishes, jaw protrusion is highly relevant to feeding kinematics and can be used to predict suction feeding ability (Cooper et al., 2010, 2017). Furthermore, it has been used extensively to explore the link between morphology and ecology in damselfish, sticklebacks, and cichlids (Cooper et al., 2017; Hulsey & García De León, 2005; Matthews & Albertson, 2017; McGee & Wainwright, 2013). Specifically, limnetic foragers have greater jaw protrusion than benthic foragers, which aids in the capture of food from the water column by increasing suction abilities (Matthews & Albertson, 2017; McGee et al., 2013; Motta, 1984). Feeding kinematics can also be influenced by plasticity in African cichlids (Bouton et al., 2002) with an algae treatment inducing a greater bite force through increased musculature attachment to the mandible, and an increased angle between the ascending and dentigerous arms of the maxilla (Bouton et al., 2002).

Phenotypic plasticity is posited to play a key role in the rapid and explosive radiation of African cichlids (Gilbert, 2021; Navon et al., 2020; Parsons et al., 2016; van Snick Gray & Stauffer, 2004; Wimberger, 1992). Given previous indications of phenotypic plasticity in African cichlids (Bouton et al., 2002; Parsons et al., 2014, 2016; van Snick Gray & Stauffer, 2004), and evidence of sexual dimorphism consistent with ESD in mandible and craniofacial shape (McWhinnie et al., 2022; McWhinnie & Parsons, 2019), we hypothesised that plastic responses would differ between sexes of Malawi cichlids. We predicted that females of both species would display reduced plasticity relative to males as they face constraints in trophic morphology due to mouth‐brooding (Tkint et al., 2012). This form of maternal care, whereby females hold eggs and larvae for a period of a few weeks is practised by many cichlids, including Malawi cichlids. We also predicted that a limnetic diet would result in greater jaw protrusion, along with associated functional changes (shorter retroarticular and longer inter‐opercular links) that would differ from the benthic treatment (Hu & Albertson, 2014; Matthews & Albertson, 2017; McGee et al., 2013; Westneat, 1995). To test these predictions, we performed a foraging experiment using two focal species; *Tropheops* “Red Cheek” (TRC) and *Labeotropheus fuelleborni* (LF). TRC is an algal grazer and is the immediate neighbour of LF in the morphospace of the Malawi radiation in terms of craniofacial shape (Cooper et al., 2010), but it has a much narrower mandible relative to LF (Parsons et al., 2011), which may impact maternal care capacity. TRC feeds by plucking targeted threads of algae rather than the scraping mode employed by LF (Albertson, 2008; Albertson & Pauers, 2018). Lever mechanics measurements of the mandible suggest that *Tropheops* species differ functionally from LF by possessing a higher KT coefficient (Holzman & Hulsey, 2017). Broadly, this investigation considered how multiple levels of biological variation can contribute toward variations that in turn could influence broader patterns of adaptive divergence.

## 2 MATERIALS AND METHODS

### 2.1 Fish husbandry and rearing

Stock cichlids were obtained from the wild and purchased through the pet trade. At the University of Glasgow, they were kept in 100 L aquariums and monitored for breeding and egg holding. Cichlid broods were collected for LF and TRC from females at 3 days post fertilisation (dpf). A total of 101 fish from 10 broods were collected for TRC, and 115 fish from four broods were collected for LF. Each brood was raised separately in a 1 L conical flask with 1–2 drops of methylene blue to prevent fungus growth and the embryos were kept aerated. At around 20 dpf, the yolk was nearly fully absorbed, and each brood was moved into a separate 25 L tank to feed independently. After a further 4–6 weeks, the broods were divided and moved into larger (~125 L) tanks as part of a mixed family group. In total there were four treatment tanks for each species; two for the benthic treatment and two for the limnetic. Each tank contained the same enrichment and used the same water supply. To limit potential effects from density, each family was divided approximately equally into four treatment tanks containing between 22 and 26 fish.

### 2.2 Diet treatment experiment

To test the impact of different foraging and biomechanical demands on morphology, treatment groups were fed either limnetic or benthic food based on previous methods (Parsons et al., 2014, 2016). The nutritional content of food was kept similar to limit the possibility of nutritional effects on morphological plasticity (Wimberger, 1992). The limnetic treatment, given to half of the groups, consisted of a ground mixture of flake food, algae wafer and freeze‐dried daphnia, which was then sprinkled into the water column to elicit suction feeding. The benthic treatment, given to the other half of treatment groups was the same mixture but air dried on to lava rocks. During feeding, these rocks were placed at the bottom of the tank to elicit a biting mode of feeding. Each treatment tank was fed twice daily until satiation (morning and afternoon feeds) for approximately 6–7 months until fish were within the size range of a mature cichlid (approximately 4–8 cm standard length (SL)) and sexual dimorphism in colouration and spawning activity had begun. Smaller fish were excluded from downstream analysis as they were difficult to dissect (*n* = 4). All fish were euthanised following UK Home Office Schedule 1 guidelines, labelled, and fixed in 10% neutral buffered formalin (NBF). Fish were sexed using colouration and venting (Moore & Roberts, 2017). Dissection of internal anatomy was also conducted to confirm sex.

### 2.3 Morphometrics

**FIGURE 1:**
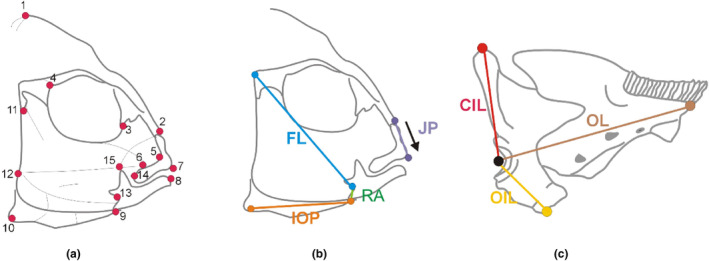
The landmarks selected for morphometrics based on functional and ecological relevance (Cooper et al., 2010; Parsons et al., 2011, 2016), as well as functional linear traits on the head and mandible. Landmarks (a) represent the following anatomical locations: (1) Dorsal end of the occipital crest. (2) Posterior tip of the ascending arm of the premaxilla. (3) Anterio‐ventral point of eye socket. (4) Posterio‐ventral point of eye socket. (5) Maxillary‐palatine joint. (6) Muscle insertion on the maxilla. (7) Tip of the tooth on the pre‐maxilla. (8) Tip of the tooth on the mandible. (9) Retroarticular of the mandible. (10) Posterio‐ventral corner of preopercular bone. (11) Origin point of muscle insertion on the pre‐opercular. (12) Posterio‐ventral corner of muscle origin. (13) Articular‐quadrate joint. (14) Maxillary‐articulation joint. (15) Muscle insertion on the articular process of the mandible. The functionally relevant traits measured on the head and mandible of cichlids. For the head in panel b, three traits were measured (JP = jaw protrusion; IOP = interopercular link; RA = retroarticular process). Here, the fixed link (FL in blue) was used as a ratio to factor out size for the IOP (in orange) and the RA (in green) of the opercular four‐bar linkage. In panel c, the cichlid mandible is depicted with measures of mechanically relevant traits, the outlever (OL in brown), the closing‐in lever (CIL in red), and the opening‐in lever (OIL in yellow). The mechanical advantage of opening for the mandible (MAo) and for closing (MAc) were respectively derived by division of OIL/OL, and CIL/OL.

Following fixation, the craniofacial region was dissected to reveal musculature and allow landmarks to be collected for geometric morphometrics. Craniofacial landmarks (Figure 1) were selected based on previous work (Parsons et al., 2016; Rundle, 2002) to ensure that they were relevant to the evolution and functional anatomy of cichlids. Fish were secured to a wax dish with a scale and ID tag and photographed laterally from a fixed distance, with their mouth closed, using a mounted Canon EOS 1100D camera (Canon (UK), Surrey).

For landmark digitisation, the tps suite of software was used (available at: http://life.bio.sunysb.edu/ee/rohlf/software.html). First, a tps file linking photographs was created from all photographs using tpsUtil. Prior to digitisation and to reduce intra‐observer variability, the ID tags were removed from the images and the photographs were randomised using tpsUtil. Digitisation of landmarks was conducted using tpsDig2 with a scale factor measured for each image. Following digitisation, the tps files were analysed in R version 4.0.4 (R Core Team, 2021) using the geomorph package (Adams & Otárola‐Castillo, 2013) unless stated otherwise. Before any analysis could take place, the landmarks were subjected to a Procrustes superimposition that translated, rotated and scaled specimens to a common centroid size using *gpagen* (Zelditch et al., 2004). Procrustes coordinates were then used for all downstream analyses.

Because size/shape relationships could vary across species, we tested for the potential influence of allometry, to expose biologically relevant differences in shape. This was necessary before further modelling (see below) could occur. Therefore, as our data indicated that allometric slopes differed between species (when tested with a Procrustes ANOVA using *procD.lm*) a common allometric regression was not applied across our samples prior to further analyses (Klingenberg, 2016).

To assess the effect of species, diet, sex, and their interactions on shape Procrustes ANOVA, using the *procD.lm* function, was conducted on the Procrustes coordinates. In particular, diet by sex interactions were of interest to test our core hypothesis that plasticity contributes to ESD through divergence in shape plasticity. Thus, our model included (in this order) sex, diet treatment, species, and the log of geometric centroid size as factors. If ESD contributes to the radiation it would likely differ across species. Thus, interactions between sex and species would indicate that sexual dimorphism had diverged between species, treatment by species interactions would indicate that plasticity had diverged between species, while the three way interaction of sex, treatment, and species would indicate that sexual dimorphism in plasticity had diverged between species. All interactions were modelled using a type 1 sum of squares approach.

Also, to further explore shape plasticity, we tested whether specimens could be classified using a priori groupings (treatment and both sexes of each treatment for each species). This was conducted through a discriminant function analysis (DFA) on all PC scores, generated by *plotTangentSpace* from the Procrustes shape coordinates, using the *lda* function in the MASS package (Venables & Ripley, 2002). To test the hypothesis that species and sexes would differ in the magnitude of plasticity, partial Procrustes distances (PPD) were calculated for each species between each treatment group as a whole and divided by sex. Differences in distances between groups were compared using TwoGroup from the IMP suite of software (available at: http://www.philadb.com/an‐behav/imp/) with 900 bootstraps (Zelditch et al., 2004). Finally, to visualise shape changes relating to diet between sexes and species, the scores obtained from the DFA were regressed on Procrustes corrected landmarks to produce deformation grids from using *shape.predictor*.

### 2.4 Measurement of functionally relevant traits

Traits of importance to fish jaw function were targeted for quantification through a second and third set of photographs. This included linear measures of the retroarticular (RA) process of the mandible, and the interopercle (IOP) link, which extends from the IOP bone to the insertion of the IOP ligament on to the RA. The IOP directly transmits motion to the mandible through the interoperculomandibular ligament that inserts on to the posterior point of the RA of the mandible (Westneat, 1990) (Figure 1). Therefore, both traits form two primary links in the teleost opercular four‐bar linkage model with lengthening and shortening of these links being highly relevant for functional predictions (Hu & Albertson, 2014; Westneat, 1990). Generally, a short RA and long IOP leads to faster jaw rotation and a reduction in the mechanical advantage (MA) of the jaw and is favourable for suction feeding (Hu & Albertson, 2014). Conversely, a long RA and short IOP leads to a higher MA, but with reduced jaw opening speed, and usually occurs with a biting mode of feeding (Barel, 1983; Hu & Albertson, 2014; Westneat, 2003).

To assess the plasticity of the IOP and RA in response to foraging treatment, fish were taken through a clearing and staining protocol following Pothoff (Hulsey & Wainwright, 2002). First, the fish were stained with alizarin red at a ratio of 1:40 in 1% potassium hydroxide solution (KOH) to highlight areas of bone for identification of the IOP link and the RA. After staining, fish were stepped through a series of KOH and glycerol changes following Pothoff (Hulsey & Wainwright, 2002) to clear excess stain and then photographed with the mouth closed. As before, landmarks were placed on photographs to identify inter‐landmark distances. Additionally, landmarks comprising the fixed link of the opercular four‐bar were also measured (Figure 1) and used to standardise for size. Both the RA and IOP were calculated as a ratio of the fixed link following Hulsey and García De León (Hulsey & García De León, 2005) as this is a relevant method of removing size variation from the measurements of links in a four‐bar mechanism (Westneat, 1990). The ratios were then subjected to separate ANOVA using species, treatment, sex and their interactions as factors.

Additionally, to assess jaw function fish were photographed from the left lateral view with their mouth open (Matthews & Albertson, 2017; McGee et al., 2013). The jaw was opened by first securing the fish on a wax dish, and then using forceps to open the jaws by using a probe to press onto the ventral side of the neurocranium (McGee et al., 2013). These lateral photographs with the mouth open and upper jaw protruded were captured and two landmarks were digitised on each photo, representing the length of jaw protrusion (the length of the dentigerous arm) (Matthews & Albertson, 2017). This process of performing manual jaw protusion, photographing, and measuring was repeated three times, and while little to no variation was observed, we used the most complete protrusion measurement for our analysis. Although not a direct measure of kinematics, this measure did reflect a dynamic morphological system, separating it from facial morphology and making it more kinematic in nature (Matthews & Albertson, 2017). All linear distances were calculated in geomorph using the *interlmkdist* function. The maximum linear distance for each individual was then standardised for size using a linear regression against the SL. The size‐standardised residuals were then used to test the effect of species, treatment, sex and their interaction using ANOVA.

To further assess biomechanical variation in the ability of the jaw to function the mandible was photographed for each specimen. Specifically, each mandible was partially dissected from each fish to expose the left lateral view, which was photographed using a Leica M165 FC stereomicroscope mounted with a Leica DFC 450C digital camera and using associated LAS v4.4 software (Leica Microsystems). For each mandible, we placed landmarks that allowed us to quantify lever ratios for the mechanical advantage of opening (MAo) and closing (MAc) using the ratios of the opening‐in lever and the closing‐in lever to the outlever (see Figure 1; Hulsey & García De León, 2005; Parsons et al., 2016). Variations in MAo and MAc were then modelled in separate ANOVA models to test for the effect of species, sex, treatment and their interaction.

## 3 RESULTS

### 3.1 Morphological plasticity

**FIGURE 2:**
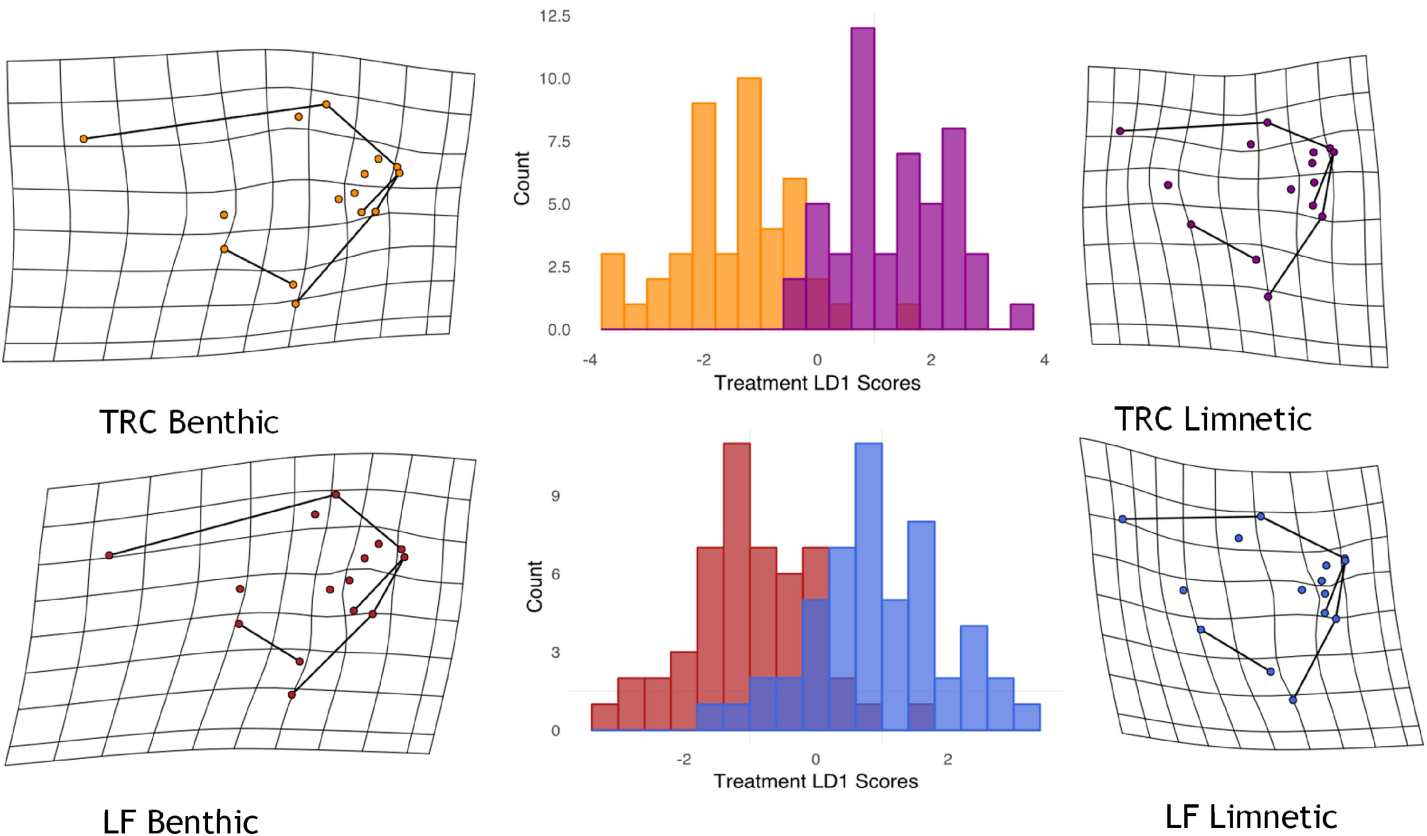
Frequency histograms displaying the classification rate of diet treatment with accompanying deformation grids depicting associated shape variation for each species. For *Tropheops* “Red Cheek” (TRC), the benthic specimens are represented in orange and limnetic in purple while for *Labeotropheus fuelleborni* (LF) the benthic specimens are represented in red and the limnetic are in blue.

Both species (TRC *n* = 94, LF *n* = 101) displayed phenotypic plasticity in response to foraging treatments. However, our Procrustes ANOVA showed that plasticity only differed marginally between species (Table 1), while no other significant interactions occurred. In line with this marginal interaction between treatment and species, the discriminant function models indicated that 91% of the benthic and 92% of limnetic treatment TRC specimens were classified correctly whereas classification rates were lower for LF where 88% of benthic and 84% of limnetic specimens were correctly classified (Figure 2). Nonetheless, the magnitudes of plasticity were similar between species as indicated by PPDs (95% CI of difference between species = −0.013 to 0.012) suggesting localised anatomical responses unique to each species. Indeed, for both species the benthic treatment resulted in a steeper face and shorter mandible relative to the limnetic treatment specimens, although in TRC there was a greater change in the mandible, as well as an expansion of the occipital crest under benthic conditions (Figure 2).

**TABLE 1:** Summary output from the Procrustes ANOVA model used to assess the phenotypic plasticity and sex effects on craniofacial shape across cichlid species.

Factors	df	Sum Sq	Mean Sq	*R* Sq	*F*	*Z*	*p*
Sex	1	0.029	0.029	0.029	8.016	4.610	.001**
Treatment	1	0.040	0.040	0.040	10.974	4.998	.001**
Species	1	0.214	0.214	0.214	58.978	7.695	.001**
logCS	1	0.026	0.026	0.026	7.154	5.204	.001**
Sex:Treatment	1	0.003	0.003	0.003	0.754	−0.513	.694
Sex:Species	1	0.004	0.004	0.004	0.988	0.228	.414
Treatment:Species	1	0.006	0.006	0.006	1.634	1.361	.082*
Sex:Diet:Species	1	0.003	0.003	0.002	0.880	−0.075	.527
Residuals	186	0.674	0.004	0.675			

*Note*: *p < .05; **p < .001.

**FIGURE 3:**
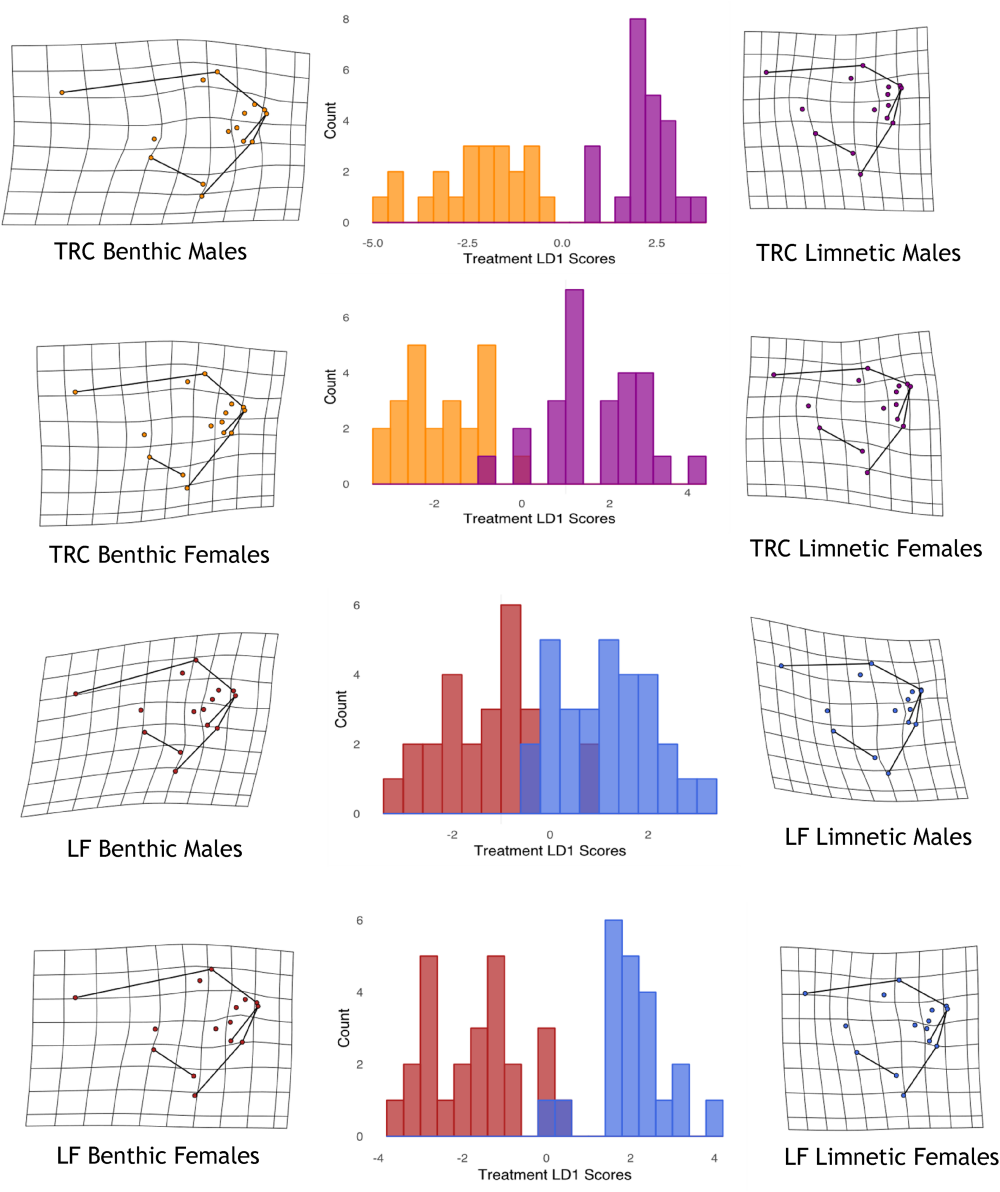
Frequency histograms for sex derived from discriminant function analysis (DFA) models using treatment as a grouping variable for each sex and species. For *Tropheops* “Red Cheek” (TRC), the benthic specimens are represented in orange with the limnetic in purple. For *Labeotropheus fuelleborni* (LF) the benthic specimens are represented in red with the limnetic in blue. The shape changes associated with each DFA model are depicted with deformation grids to the right and left of the histograms.

Although sexual dimorphism occurred in craniofacial shape this did not interact with species, while plasticity also showed no interaction with sex suggesting that ESD is not promoted by shape plasticity (Table 1). Classification success for sex based on DFAs was similar for each sex and species regardless of treatment (LF = 92% of benthic males, 93% of limnetic males and 96% of benthic females and 95% of limnetic females; TRC = 96% of benthic females, 92% of limnetic females, 100% of benthic males and 100% of limnetic males). In addition, the PPDs showed no differences for sex (Table 2). Shape changes were similar for each sex undergoing the benthic and limnetic treatments with benthic fish having a steeper profile than the relatively sloping profile of the limnetic treatment fish (Figure 3).

**TABLE 2:** The partial procrustes distance and associated 95% confidence intervals that occur between groups after 900 bootstraps. Comparisons between each treatment and sex are provided for both cichlid species.

Species	Groups	Partial Procrustes distance	95% CI
LF	Benthic and Limnetic	0.031	0.025–0.043
	Benthic Males and Limnetic Males	0.033	0.025–0.048
	Benthic Females and Limnetic Females	0.031	0.025–0.047
TRC	Benthic and Limnetic	0.032	0.026–0.042
	Benthic Males and Limnetic Males	0.034	0.026–0.053
	Benthic Females and Limnetic Females	0.033	0.028–0.046

### 3.2 Plasticity of functional traits

**FIGURE 4:**
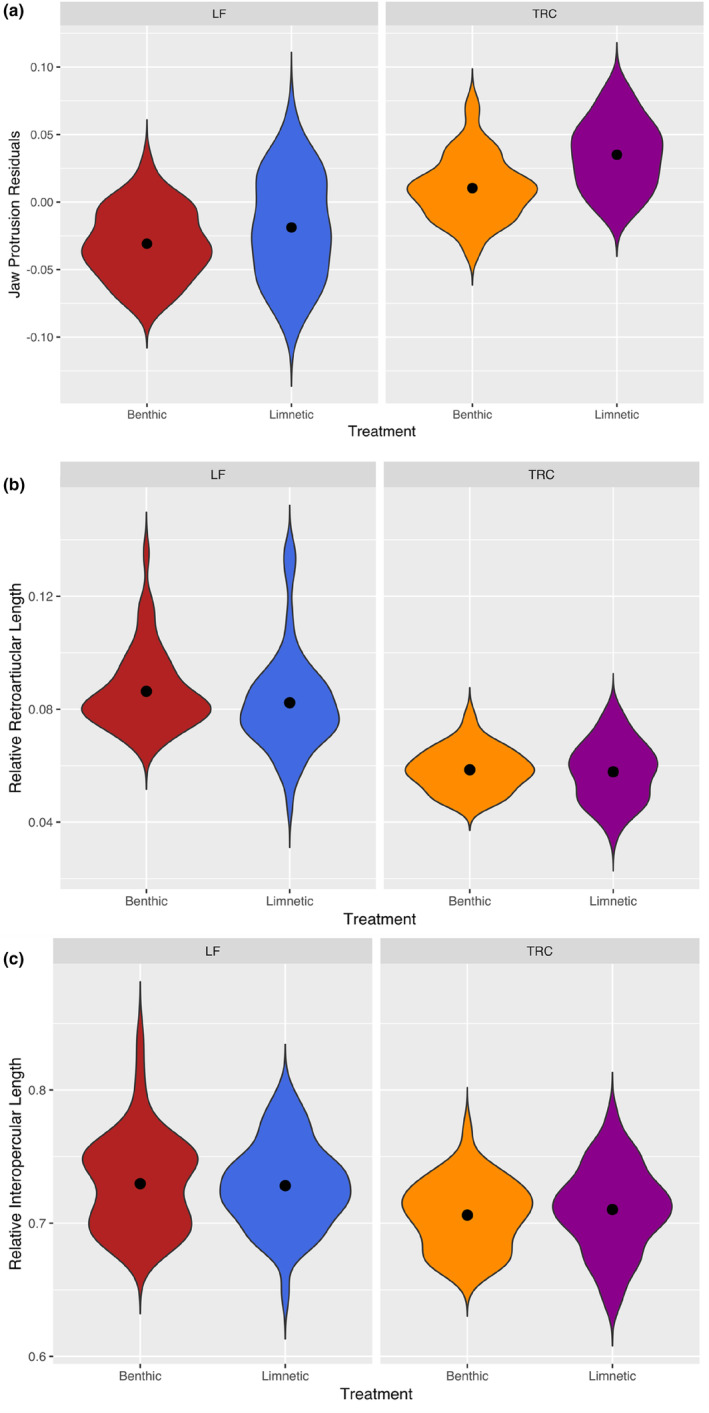
Comparison of functional morphological traits between species (*Tropheops* ‘red cheek’ = TRC, and *Labeotropheus fuelleborni* = LF) and benthic and limnetic foraging treatments. In panel (a) jaw protrusion residuals are provided for each treatment and species (*n* = 177), while for (b) relative retroarticular (RA) length (cm) for each treatment for both species (*n* = 193) and (c) relative interopercular (IOP) length (cm) for each treatment for both species (*n* = 193).

For all functional traits, the two species differed but only jaw protrusion and MAo revealed foraging treatment effects. Notably, there was an interaction between species and treatment for MAo (Table 3, Figure 4). For both species, jaw protrusion was greater for the limnetic treatment than for the benthic, but this difference did not appear as pronounced for LF where there was considerable overlap between the two treatments (Figure 4, Table 3). Finally, sex did interact with treatment for MAc, indicating sexual dimorphism in its plasticity.

**TABLE 3:** The results of ANOVA models examining functional traits.

Trait	Factors	df	Sum Sq	Mean Sq	*F*	*p*
Jaw Protrusion	Species	1	69.4362	69.4362	122.425	2 × 10^−16^***
	Treatment	1	10.1260	10.1260	17.853	4 × 10^−5^***
	Sex	1	0.0163	0.0163	0.029	.866
	Species:Treatment	1	1.2100	1.2100	2.133	.146
	Species:Sex	1	0.0007	0.0007	0.001	.972
	Treatment:Sex	1	0.0483	0.0483	0.085	.771
	Species:Treatment:Sex	1	1.0189	1.0189	1.797	.182
	Residuals	171	96.9865	0.5672		
Relative RA	Species	1	0.0328	0.0328	187.786	2 × 10^−16^***
	Treatment	1	0.0005	0.0005	2.774	.098
	Sex	1	0.0000	0.0000	0.012	.912
	Species:Treatment	1	0.0001	0.0001	0.681	.410
	Species:Sex	1	0.0001	0.0001	0.269	.604
	Treatment:Sex	1	0.0000	0.0000	0.010	.921
	Species:Treatment:Sex	1	0.0002	0.0002	1.081	.300
	Residuals	185	0.0323	0.0002		
Relative IOP	Species	1	0.0215	0.0215	21.656	6 × 10^−6^***
	Treatment	1	0.0000	0.0000	0.033	.857
	Sex	1	0.0018	0.0018	1.827	.178
	Species:Treatment	1	0.0003	0.0003	0.285	.594
	Species:Sex	1	0.0002	0.0002	0.187	.666
	Treatment:Sex	1	0.0000	0.0000	0.000	.991
	Species:Treatment:Sex	1	0.0002	0.0002	0.163	.687
	Residuals	185	0.1836	0.0010		
Mandible MAc	Species	1	0.1842	0.1842	59.045	9 × 10^−13^***
	Treatment	1	0.0084	0.0084	2.705	.102
	Sex	1	0.0041	0.0042	1.330	.250
	Species:Treatment	1	0.0003	0.0003	0.084	.772
	Species:Sex	1	0.0076	0.0077	2.451	.119
	Treatment:Sex	1	0.0172	0.0172	5.518	.020*
	Species:Treatment:Sex	1	0.0000	0.0000	0.006	.937
	Residuals	185	0.5772	0.0031		
Mandible MAo	Species	1	0.0446	0.0446	21.141	8 × 10^−6^***
	Treatment	1	0.0087	0.0087	4.111	.044*
	Sex	1	0.0029	0.0029	1.374	.243
	Species:Treatment	1	0.0092	0.0092	4.361	.038*
	Species:Sex	1	0.0034	0.0034	1.590	.209
	Treatment:Sex	1	0.0030	0.0030	1.403	.238
	Species:Treatment:Sex	1	0.0077	0.0077	3.661	.057
	Residuals	185	0.3902	0.0021		

*Note*: This included size‐corrected jaw protrusion residuals (*n* = 178), relative retroarticular (RA) length (*n* = 192) and relative interopercular (IOP) length (*n* = 192), and mandibular mechanical advantage closing (MAc) and mandibular mechanical advantage opening (MAo). Asterisks denote statistically significant *p* values. *p < .05; ***p < .001.

## 4 DISCUSSION

We tested whether phenotypic plasticity differed between sexes across two species of African cichlids. In both species, sex differences in craniofacial shape occurred but were not related to plasticity. This contradicted our prediction that males would display more plasticity than females. Therefore, for head shape within these cichlids phenotypic plasticity may not be important for ESD despite its role in the Malawi radiation and more conventional types of adaptive divergence in fishes (Pfennig et al., 2010; Potthoff, 1984). However, ESD was indicated for MAc through an interaction between sex and treatment. Given that MAc is a well described functional trait this suggests that such sexual dimorphism in plasticity could have ecological implications. Also, given that treatment alone did not affect MAc, this plasticity may only have the opportunity to evolve through a linkage with sex. Indeed, we suggest that ESD could be a case of adaptive divergence that is strongly influenced by genetically determined processes, possibly through sex‐linked variation (Parsons et al., 2015), but in some cases can include sex‐based plasticity. This highlights the need to investigate adaptive divergence at multiple levels of organisation including under different environments to gather a wholistic view for how variation arises.

Both cichlid species exhibited phenotypic plasticity in craniofacial shape, and divergence in plasticity between species was suggested. While plasticity marginally differed between species the effect size was relatively small in our model (interaction term accounting for 0.77% of the variation). Nonetheless, the lower magnitudes of plasticity indicated for LF (i.e. weaker grouping based on foraging treatments) could be due to their highly specialised phenotype. Specifically, their phenotype enables efficient handling of mechanical stress (McWhinnie et al., 2022) and may in turn reduce the opportunity for bone remodelling (i.e. plasticity). This could have broader implications as increases in specialisation are predicted to lead to reductions in phenotypic plasticity during adaptive divergence (Parsons et al., 2014; Skúlason et al., 2019; Skúlason & Smith, 1995). However, this prediction relies on costs of plasticity as a driver of developmental canalization. Alternatively, we suggest that the evolution of functional efficiency could itself also provide a mechanism for such a phenomenon. In other words, in some cases such as where mechanical stress is an outcome of adaptive variation, this could feedback to result in a more canalised phenotype (Gilbert, 2021).

Traits associated with jaw function were plastic in both species indicating potential adaptive implications for responses to foraging treatments. Specifically, benthic treatment fish had a shorter jaw protrusion than limnetic‐reared fish, a finding in line with a morphology that should incur a relatively reduced ability to suction feed in benthic‐reared fish (Motta, 1984; Waltzek & Wainwright, 2003). Between species TRCs possessed greater jaw protrusion than LFs (Figure 4) as would be expected. However, there was a lack of sexual dimorphism in plasticity for jaw protrusion. Given that variation in jaw protrusion is arguably one of the most important traits involved with adaptive divergence in fishes, and that this trait has been proposed as a key innovation in the evolution of vertebrate suction feeding, this could have substantial evolutionary consequences (Conith et al., 2018). Indeed, for three‐spine sticklebacks sexual dimorphism in jaw protrusion is prevalent within some populations (McGee & Wainwright, 2013). ESD through variation in jaw protrusion has in turn been suggested to enable sticklebacks to quickly adapt into limnetic and benthic ecomorphs when colonising new habitats. Given our findings, it may be that such sexual dimorphism does not always represent the same underlying mechanisms found in wider patterns of adaptive divergence. Instead, ESD may sometimes provide an alternate form of divergence from a developmental and genetic perspective, that phenotypically resembles wider patterns of divergence between species but does not initiate further adaptive divergence (Parsons et al., 2015). In other words, ESD may allow a population to persist in a new habitat by allowing for partitioning of habitats and resources, but it may not initiate the direction of more conventional ecological divergence. For most of our measured traits, our findings suggest that plasticity is excluded from ESD further reinforcing this idea. To address these ideas, future research investigating whether the mechanisms of divergence at the population‐level, and sexual dimorphism are exclusive would be especially enlightening.

However, the interaction between sex and treatment for MAc suggests that plasticity can have a role in ESD, although one that is anatomically localised. This trait should be strongly associated with bite force and thus play a role in foraging efficiency for both these algal grazing species. While plasticity is often considered as a means for establishing the directions of adaptive divergence (Potthoff, 1984; West‐Eberhard, 2003), its association with sex in this context is not usually considered. Our findings suggest an interaction that is general across both species, which indicates it may not play a role in species divergence. Nonetheless, sexual dimorphism in MAc plasticity suggests that it could be linked to sex determining loci but previous genetic investigation of morphological plasticity in a hybrid cross of LF and TRC does not support this idea (Parsons et al., 2016). Thus, if the genetic basis of sexually dimorphic plasticity occurs in areas of the genome that are not involved in sex determination it could provide a means for ESD to coexist with conventional forms of adaptive divergence. Indeed, it has been predicted that both types of adaptive divergence respresent two sides of the same ecological coin that should not readily co‐exist (Bolnick & Doebeli, 2003). However, models suggest that the genetic basis of male and female ecological traits greatly affects whether a population will undergo speciation. Indeed, these models suggest that a population with a large capacity for sexual dimorphism is less likely to undergo speciation (Bolnick & Doebeli, 2003). Thus, it is notable that sex alone did not effect our functional traits, only one trait displayed an interaction between sex and plasticity, and that previous study shows plasticity in cichlid functional traits does not localise to the sex determining region. This indicates a genetic architecture in Malawi cichlids primed for adaptive divergence via speciation. At the same time, evidence is emerging that ESD and conventional adaptive divergence can co‐exist in some populations (Cooper et al., 2011).

While our data does not point towards widespread sex‐based differences in plasticity, there was clear evidence for sexual dimorphism in shape. This agrees with the expectation that mouthbrooding would act as a constraint for females. This is because mouthbrooding likely involves a different set of functional requirements that may be at odds with foraging. For example, given that “biting” requires more force and mechanical advantage, the associated larger jaw muscles could reduce space available for mouthbrooding (Tkint et al., 2012). In line with this, the only trait that did show sexual dimorphism in plasticity was MAc, a trait which should be intimately linked with jaw muscle size and result in functional consequences. Indeed, finite element modelling of the mandible during biting in these species has suggested superior bite force transmission in males (Tkint et al., 2012). Such consequences could drive different adaptive strategies between sexes in African cichlids and set limits on the range of phenotypes possible within their adaptive radiations. However, without much involvement from sexually dimorphic plasticity such limits may not exist in a way that creates a trade‐off between ESD and the broader adaptive radiation which is thought to involve plasticity (Parsons et al., 2016).

## 5 CONCLUSIONS

For Lake Malawi cichlids, it has been suggested that plasticity is still actively evolving (Parsons et al., 2016). However, our evidence suggests that plastic responses are not usually sexually dimorphic in morphological traits and therefore may not be important for the maintenance of ecological divergence between the sexes. Given the differing selection pressures sexes likely face, and the trend for females to possess more of a “suctioning” phenotype suited to carrying eggs, it would be of interest to assess whether the absence of a sexually dimorphic plastic response has negative consequences for females and mouthbrooding (Parsons et al., 2015; Tkint et al., 2012), or whether sexually dimorphic MAc plasticity can contribute to reproductive traits such as the number of eggs per brood. While our results suggest that plasticity is likely not for maintaining ESD itself, it may be that the underlying genetic basis of trait variation can enable ESD to co‐exist with other forms of ecological divergence. More mechanistic considerations would be insightful for adaptive divergence, especially study systems where there is strong evidence of ESD alongside conventional adaptive divergence.

## AUTHOR CONTRIBUTIONS

**Kirsty McWhinnie:** Conceptualization (equal); data curation (lead); formal analysis (lead); funding acquisition (lead); investigation (lead); methodology (lead); visualization (lead); writing – original draft (lead). **Deepti Negi:** Formal analysis (supporting); methodology (supporting). **K. Elizabeth Tanner:** Supervision (equal); writing – review and editing (equal). **Kevin J. Parsons:** Conceptualization (equal); formal analysis (supporting); funding acquisition (supporting); methodology (supporting); resources (equal); supervision (lead); writing – review and editing (equal).

## Data Availability

All data will be made available on dryad before publications. All data will be made available through deposition at Dryad https://doi.org/10.5061/dryad.1g1jwsv35.
